# Early Childhood Behavioral and Social-Emotional Development Among Asian Indian, Filipino, and Korean Families in the United States: A Pilot Study

**DOI:** 10.3390/children13020256

**Published:** 2026-02-12

**Authors:** Soyang Kwon, Nidhi S. Gopagani, Lin Bian, Milkie Vu

**Affiliations:** 1 Buehler Center for Health Policy and Economics, Feinberg School of Medicine, Northwestern University, Chicago, IL 60611, USA; nidhi.gopagani@northwestern.edu; 2 Department of Psychology, University of Chicago, Chicago, IL 60637, USA; linbian@uchicago.edu; 3 Department of Preventive Medicine, Northwestern University, Chicago, IL 60611, USA; milkie.vu@northwestern.edu

**Keywords:** acculturation, parenting practices, physical activity, screen time, sleep, wellbeing, Asian Americans, ActiGraph accelerometers

## Abstract

Background/Objectives: Socio-cultural adversities and health disparities across Asian American origin groups remain understudied, particularly in early childhood. This limits the development of culturally responsive prevention and intervention strategies. A family-based Asian American epidemiologic study is essential to address these gaps and to inform tailored solutions. As an initial pilot effort, this pilot study was designed primarily to assess feasibility and generate preliminary data to inform future hypothesis-driven, large-scale epidemiologic research. The study objectives were to evaluate the feasibility of a remote study protocol and to collect preliminary data on child development and parental factors among Asian Indian, Filipino, and Korean American families with young children. Methods: A remote pilot study was conducted in 2024–25 among 48 mother–father–child (age 1–4 years) triads residing in Illinois, including 18 Asian Indian, 12 Filipino, and 18 Korean American mothers. Parents completed an online survey, and children wore an ActiGraph accelerometer on their hips. Analyses were conducted to describe child development, parental experiences, and parenting practices among the three ethnic groups. Results: Of the 48 mothers, 29 (60%) were US-born, and all but 1 had at least a bachelor’s degree. All parent pairs completed the survey, whereas only 34 children (71%) provided valid accelerometer data. Disaggregated data showed that, compared to children of Asian Indian mothers, children of Filipino mothers had higher daily screen time (*p* < 0.10) and higher sleep problem scores (*p* < 0.05), and children of Korean mothers had higher child–caregiver interaction scores (*p* < 0.05). Across all three groups, more favorable parenting practices were associated with lower sleep problem scores, higher wellbeing scores, and higher child–caregiver interaction scores (*p* < 0.01). Conclusions: The remote study protocol was generally feasible; however, child compliance with hip accelerometer wear was suboptimal. Preliminary data revealed differences in children’s physical behaviors and social-emotional development across Asian ethnic groups. A full-scale study should enhance the engagement of socioeconomically diverse families and refine wearable data collection methods to improve data representativeness and completeness.

## 1. Introduction

Asian Americans represent the fastest-growing racial group in the United States (US), with a 35.5% population increase between 2010 and 2020 [1]. Despite this rapid growth, little attention has been given to the socio-cultural stressors experienced by this population, including acculturative stress and racial discrimination, which are linked to elevated risks for mental health problems and chronic conditions including diabetes and cardiovascular disease [2,3]. Moreover, Asian Americans are a highly heterogeneous group, yet health data are often reported in aggregated form under a single pan-ethnic category. This practice masks critical differences in risk profiles across origin-specific groups [4,5,6,7,8,9,10]. For instance, while Asian Americans overall appear to have a lower cardiovascular risk than their non-Hispanic White counterparts, this pattern is largely driven by East Asian groups (e.g., Korean), whereas South Asian groups (e.g., Asian Indian) face a substantially higher cardiovascular risk [5,6]. Such data aggregation obscures origin group-specific vulnerabilities and undermines efforts to develop culturally responsive prevention and intervention strategies [11,12,13].

Recent initiatives underscore the importance of collecting and reporting disaggregated health data for Asian American populations [14]. One notable initiative is the Multiethnic Observational Study in American Asian and Pacific Islander Communities (MOSAAIC), a large-scale national cohort study designed to collect epidemiologic data from adults across diverse Asian origin groups [15]. While MOSAAIC will provide valuable data into adult health, it does not capture childhood development and health. Understanding the developmental trajectories in early childhood and intergenerational (parent–child) influences is essential for informing tailored strategies to promote positive childhood experiences (PCEs) and foster well-being in the next generation of Asian Americans. Data from the National Survey of Children’s Health (NSCH) show that the prevalence of flourishing, an indicator of positive psychological wellbeing, was the lowest among Asian American children under 5 years (72.6%) compared to Hispanic (76.7%), non-Hispanic Black (79.5%), and non-Hispanic White (87.2%) children [16]. Asian American children in this age group were also less likely to have regular bedtime and more likely to have excessive screen time than their non-Hispanic White peers [17]. However, the absence of Asian origin information limits the ability to identify which origin groups are most at risk.

To address these knowledge gaps, family-based epidemiologic studies that include multiple Asian origin groups are needed. As a foundational step toward launching such an epidemiologic study, this pilot study was designed primarily to assess feasibility and generate preliminary data on child physical behaviors (physical activity [PA], screen time, and sleep) and social-emotional development, and parental experience and parenting practices among Asian Indian, Filipino, and Korean American families with children aged 1–4 years. We selected these three origin groups to represent the regional (South, Southeast, and East Asia) and cultural heterogeneity of the Asian diaspora based on their distinct physical behaviors reported in the literature [18,19,20,21,22,23] and the research team’s established community partnerships that support recruitment feasibility. These groups were also chosen among the six largest Asian origin populations in the Chicago area (Asian Indian, Chinese, Filipino, Korean, Vietnamese, and Japanese, in order). This study was guided by a socioecological framework that emphasizes how individual, family, and broader sociocultural contexts jointly shape early childhood behavioral and social-emotional development. The objectives of this pilot study were to evaluate the feasibility of a remote study protocol and to collect preliminary data on child development and parental factors among Asian Indian, Filipino, and Korean American families with young children.

## 2. Materials and Methods

### 2.1. Study Design and Sample

We conducted a cross-sectional pilot study. Participants were mother–father–child triads. Eligibility criteria for mothers included (1) self-identifying as Asian Indian, Filipino, or Korean origin, (2) 18 years or older, (3) having lived in the US for at least 10 years, (4) having a child aged 1–4 years old, (5) living with the child at least 50% of the time, (6) residing in Illinois, and (7) speaking English, Hindi, Korean, Telugu, or Urdu. Language eligibility reflected the linguistic capacity of the research team. To guide future planning, we recorded the number of otherwise eligible families excluded due to language eligibility. Eligibility criteria for children included being 1–4 years of age and having no medical conditions that make it difficult or impossible to move around (e.g., impairments in walking). Only one child per family was eligible. Eligibility criteria for fathers were living with the child at least 50% of the time and speaking English, Hindi, Korean, Telugu, or Urdu. Families were enrolled if all three members met their respective eligibility criteria. The study protocol was approved by the Ann & Robert H Lurie Children’s Hospital Institutional Review Board (IRB#2024-6829). Parental permission and informed consent were obtained from parent participants.

### 2.2. Study Setting and Recruitment

Participant recruitment was conducted between May 2024 and February 2025 using multiple recruitment strategies, including community outreach, collaboration with a community consultant, and use of a healthcare patient database. Leveraging municipal demographic data and the research team’s community networks, we identified and engaged with organizations and community areas serving Asian Indian, Filipino, and Korean populations. Recruitment flyers were distributed in multiple languages across diverse community-based settings (e.g., South Asian, Filipino, and Korean community organizations, pediatric offices, schools, churches, childcare centers, public libraries, grocery stores). We also hired a community liaison to South Asian families in a local school district to serve as a community consultant. In addition, we identified potentially eligible children (aged 1–4 years) and mothers (self-identified as Asian) from a single healthcare patient database and sent a recruitment email containing study details and a link to a REDCap-based online screening form.

### 2.3. Outcomes

Health Behaviors: Guided by the World Health Organization’s (WHO) 24 h movement framework for young children [24], we assessed PA, sedentary behavior, and sleep. Mothers were instructed to assist their child in wearing an ActiGragh wGT3X-BT accelerometer (Pensacola, FL, USA) for 24 h over 6 days and record a wear log. To generate physical activity (PA) metrics, we extracted data collected between 6 a.m. and 10 p.m. [25,26,27]. After excluding non-wear periods [28,29,30,31], a valid wear day was defined as ≥480 wear minutes during the 16 h window [28,32,33]. At least 3 valid wear days were required to represent regular PA levels [34]. Daily minutes spent in moderate- and vigorous-intensity PA (MVPA) was calculated as minutes accumulated in epochs exceeding 417 vertical counts per 15 s [30,35]. Average acceleration (mg) was derived from Euclidian norm minus one gravitational unit (ENMO) data [36] using the GGIR R package (version 4.4) [37].

Parents completed the Patient-Reported Outcome Measurement Information System (PROMIS^®^) Early Childhood (EC) 8-item Sleep Problem scale [38] from which standardized T-scores for sleep problem were calculated. Parents also reported the child’s screen time with two items: “on average, how many minutes per day did your child… spend watching things on a TV (including TV, DVD/Blu-ray or videos on apps like YouTube or Netflix through the TV; spend watching videos on a tablet or smartphone?” [16,39] Responses were summed to estimate daily screen time (minutes/day) [40].

Social-Emotional Development: Social-emotional development was assessed using 5 PROMIS EC wellbeing scales (i.e., 8-item Positive Affect, 6-item Curiosity, 6-item Persistence, 5-item Flexibility, 6-item Frustration Tolerance) [41] and a social relationship scale (i.e., 5-item Child–Caregiver Interactions) [42]. Standardized T-scores were derived for each scale.

### 2.4. Exposures

The primary exposure of interest was maternal Asian origin group, determined based on self-identified Asian origin: Asian Indian, Filipino, or Korean. Other exposures included parents’ acculturation, discrimination experiences, adverse childhood experiences (ACEs), PCEs, and parenting practices.

Acculturation, which refers to a multidimensional process involving psychological, behavioral, and cultural adaptation to a new environment [43], was assessed using the 21-item Suinn-Lew Asian Self-Identity Acculturation (SL-ASIA) scale [44,45], producing a score from 1 (low acculturation [high Asian identification]) to 5 (high acculturation [high western identification]). Discrimination experience was assessed using the 5-item Everyday Discrimination Score (EDS)-short version [46,47].

ACEs refer to traumatic or stressful events that occur before age 18 years, whereas PCEs are characterized as safe, stable, and nurturing relationships and environments before 18 years of age [48]. ACEs and PCEs were measured using the standard 10-item ACE scale [49] and 7-item PCE scale [50], respectively.

Parenting practices were assessed using the 21-item Parenting Young children (PARYC) questionnaire, which asks about the frequency of three domains of parenting during the past month (i.e., support positive behavior, setting limits, and proactive parenting). Responses were recorded on a 7-point scale (1 = not at all to 7 = most of the time). Domain-specific and overall PARYC parenting scores were calculated, in which higher scores indicated more favorable parenting practices. In addition, three NSCH positive parenting items were asked: “during the past week, (1) how many days did you or other family members read to this child”? [reading]; (2) “how many days did you or other family members tell stories or sing songs to this child?” [storytelling/singing]; and (3) “on how many days did all family members who live in the household eat a meal together?” [family meals] [51]. Response options included 0 days, 1–3 days, 4–6 days, and every day.

### 2.5. Sociodemographic and Neighborhood Characteristics

Child age and sex, as well as maternal education attainment, were reported. To describe the participants’ neighborhood resources, we used the metropolitan Child Opportunity Index (COI; very low, low, moderate, high and very high), a composite index that captures neighborhood resources and conditions that matter for children’s healthy development at a census tract level [52].

### 2.6. Study Procedures

Eligibility screeners and parent surveys were administered online using the REDCap platform. Two part-time research staff were hired: one Asian Indian individual fluent in English, Hindi, Telugu, and Urdu, and one Korean individual fluent in English and Korean. Research staff reviewed screener submissions on a daily basis. The online screener incorporated an in-built CAPTCHA (Google’s Completely Automated Public Turing test to tell Computers and Humans Apart) feature to help block bots and reduce fraudulent submissions. As the study progressed, fraudulent responses were detected [53]. To address this issue, we implemented multi-layered strategies to identify fraudulent or suspicious responses. Daily screener traffic was monitored, and days with an unusually high number of completions were flagged for closer inspection. Indicators of suspicious screeners included atypical completion timestamps, extremely short completion times (i.e., <1 min), and suspicious name, email, and phone number entries. Suspicious cases underwent further verification, including video verification and address confirmation (required for mailing accelerometer packages). Questionable cases were reviewed in team discussions, and individuals determined to be fraudulent were excluded from participation.

Eligible participants identified through the screening process were contacted by email to schedule a phone or Zoom meeting. Following electronic written consent, study staff collected demographic information and provided instructions for accelerometer wear. Each parent then received an online REDcap survey link via email. The survey was only available in English due to limited research resources for translation; however, verbal translation assistance was provided upon request. An accelerometer package, including a prepaid return envelope, was mailed to participants. Upon completion of their respective study activities, each child, mother, and father received a $20 gift card in appreciation of their time and participation.

### 2.7. Statistical Analysis

All analyses were conducted in SAS 9.4 (Cary, NC, USA). A significance level was set at *p* < 0.10 (two-sided), considering the exploratory nature of this pilot study. We performed descriptive analyses and assessed normality. Bivariate analyses (i.e., *t*-tests and Analysis of Variance) were conducted to compare child outcome variables and parent exposure variables among three Asian origin groups.

Multivariable mixed-effects linear regression models were used to compare child outcomes reported by both mothers and fathers among the three Asian origin groups. Due to the relatively small sample size (n = 96) for this analysis, models included only a few key demographic and parental predictors: child age in years, child sex, parenting practice scores, and respondent identification (mother vs. father).

## 3. Results

**Figure 1:**
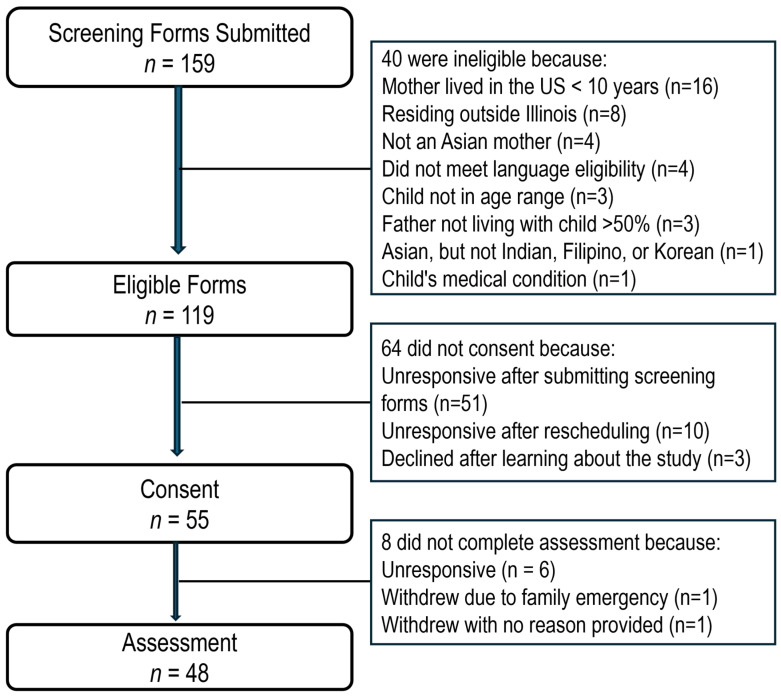
Participant flowchart.

A total of 453 screeners were filled, of which 294 (64.9%) were determined to be fraudulent during the study process. As shown in Figure 1, of the 159 valid screeners, 119 respondents were deemed eligible and 56 consented to participate. Excluding the six individuals who became unresponsive after consenting, one who withdrew due to a family emergency, and one who withdrew without providing a specific reason, 48 families participated in this study.

Among the forty-eight families, thirty-one were recruited through healthcare patient database emails, five through the community consultant, five through word of mouth, four through pediatric clinics, two through community partners’ social media, and one through a childcare center. By maternal Asian origin, 18 were Asian Indian, 12 were Filipino, and 18 were Korean. Table 1 presents participant characteristics by the three Asian origin groups. Nearly all mothers (n = 47; 98%) had a bachelor’s degree or higher and 39 (81%) resided in neighborhoods with high or very high COI.

All 48 mother–father pairs completed the parent survey, whereas 34 children (71%) provided valid accelerometer data. Among the 14 children without sufficient accelerometer data, the primary reasons were maternal refusal of accelerometer wear (n = 7), child noncompliance or insufficient wear time (n = 6), and daycare restrictions (n = 1). As shown in Table 2, when the three groups were aggregated, an average PROMIS EC Sleep T-score (mean ± standard deviation [SD] = 50 ± 9) was the same as the mean of a reference population (50 ± 10) [38]. However, disaggregated analyses showed that an average PROMIS EC Sleep T-score was lower in the Filipino group than the Asian Indian group (*p* < 0.10).

As shown in Table 3, average ACEs were higher among Filipino mothers compared to Korean mothers (*p* < 0.10). Average PCEs were lower among Filipino mothers compared to Asian Indian or Korean mothers (*p* < 0.05). The distribution of ACE and PCE items is presented in Supplementary Figure S1. PARYC parenting scores or positive parenting practices were not significantly different across the three groups. Higher parental PCE scores were associated with higher PARYC parenting scores (*p* < 0.05; Supplementary Table S1).

Multivariable mixed-effects models showed that children in the Filipino group had higher screen time and sleep problem T-scores (*p* < 0.10), compared with children in the Asian Indian group (Table 4). Children in the Korean group had higher child–caregiver Interaction T-scores, compared with children in the Asian Indian group (*p* < 0.01). PARYC parenting practice scores were negatively associated with sleep problem T-scores and positively associated with wellbeing T-scores and the child–caregiver interaction T-scores (*p* < 0.01).

## 4. Discussion

### 4.1. Main Findings

This pilot study assessed the feasibility of a remote study protocol and collected preliminary data on child development and parental factors among Asian Indian, Filipino, and Korean families with young children. While a remote study protocol was generally feasible, compliance with hip accelerometer wear among young children was suboptimal. Preliminary data demonstrated variations in early childhood physical behaviors and social-emotional development across Asian origin groups.

### 4.2. Feasibility of Recruitment and Data Collection Protocols

Prior research suggests that Asian Americans are less willing than other racial and ethnic groups to participate in health research [54], partly due to mistrust and language barriers [54,55,56]. Findings from this pilot study indicate that recruiting Asian American families with young children is feasible, but requires multifaceted outreach efforts. Of the 56 families who consented, 48 ultimately participated in the study. The majority (65%) were recruited through healthcare system outreach, suggesting that leveraging electronic health record (EHR)-based patient communication is a promising strategy for engaging Asian American parents. However, community-based methods, including engagement through a community consultant and word of mouth, also contributed to recruitment. Community engagement may be especially important to reach families underrepresented in healthcare systems [57]. A 10-year residency requirement for mothers excluded a notable proportion of interested individuals (16 out of 40 excluded individuals), suggesting that eligibility criteria such as residency duration and language requirements should be reconsidered in a full-scale study to best align with study goals and to enhance inclusivity.

This study’s remote design expanded its geographical reach and reduced participation burden for families with young children (e.g., travel time, work absences). However, the online recruitment screener encountered a large number of fraudulent or suspicious responses, with nearly two-thirds (65%) of submissions being excluded. Future remote recruitment efforts should incorporate multi-layered fraud prevention strategies (e.g., IP tracking, video verification) and adapt them over time as fraud tactics evolve [58,59,60].

Remote data collection through online surveys was largely successful: all 48 mother–father pairs successfully completed the surveys. However, child compliance with hip accelerometer wear was limited; only 34 of 48 children (71%) provided sufficient accelerometer data. Among those without accelerometer data, parental refusal (n = 7) and child noncompliance (n = 6) were the primary reasons. This suboptimal engagement in wearables’ data collection in Asian American families has been documented [61]. Our findings indicate that although mailed device protocols are logistically feasible, optimizing participation and compliance in wearable-based research among Asian American families requires intentional community trust-building and the exploration of alternative wearable implementation strategies (e.g., an alternative device).

### 4.3. Asian Mothers’ Lived Experiences

When data were aggregated across the three Asian origin groups, ACEs and PCEs reported in mother participants were lower than those reported by non-Hispanic White adults in US national datasets [48,62]. These findings align with prior national research showing that Asian adults reported lower ACEs [62] and lower PCEs [48] compared to non-Hispanic White adults. In this sample, the PCE item, “*I was able to talk with my family about feelings*” had the lowest endorsement, consistent with national data indicating similar low endorsement for “*I was able to talk with family about feelings*” and “*I had at least two non-parent adults who genuinely cared*” [48].

The lower ACEs observed could reflect genuinely lower exposure to adversity due to protective cultural, familial or community factors, such as strong family cohesion and extended family support [63]. Lower PCEs may reflect cultural norms emphasizing emotional restraint, indirect communication, and less explicit verbal expression of feelings rather than the overt expressive praise typical of Western cultures [64,65]. PCE constructs emphasize emotional support, open communication, and positive reinforcement, whereas for Asian families, positive experiences may be conveyed through instrumental support, high parental involvement, and relational commitment, even when emotional expression is more restrained [66,67]. These results may also reflect reporting bias, as cultural norms can discourage disclosure of family conflict, emotional abuse, or psychological distress, thereby limiting the recognition or reporting of certain adversities [68,69]. Overall, these findings underscore the importance of culturally sensitive interpretations of ACE and PCE measures.

Prior studies [48,62] were not disaggregated ACE and PCE data by Asian origin groups. In this pilot study, maternal ACEs and PCEs did vary across Asian origin groups. Disaggregated data revealed that Filipino mothers reported higher ACEs and lower PCEs than Asian Indian and Korean mothers. Extremely limited data are available to explain and contextualize the differences in childhood adversity and protective experiences across these origin groups, highlighting a critical need for disaggregated, culturally grounded studies in this area.

### 4.4. Asian Mothers’ Parenting Practices

This pilot study found no significant differences in the maternal parenting practices investigated among the three Asian origin groups. Notably, a higher proportion of mothers in this sample reported frequent positive parenting practices (i.e., reading to the child, telling stories/singing songs, and eating meals together), compared to findings from a national sample of US Asian parents [17]. This may be partly explained by relatively high socioeconomic status of our participants (i.e., high education attainment), as maternal socioeconomic advantage is associated with positive parenting practices [70,71].

Prior research has linked greater parental ACEs to higher parenting stress, harsher discipline, and poorer parent–child interactions, which elevate the risk of the offspring’s developmental delays and internalizing or externalizing problems [72,73,74,75,76]. In contrast, greater parental PCEs have been associated with more nurturing parenting attitudes and lower harsh parenting beliefs, potentially buffering the negative effects of ACEs on parenting behaviors [77,78]. Consistent with these previous studies [77,78], the present study found that PCE scores were positively associated with parenting practice scores among Asian mothers, suggesting the potential protective role of mothers’ PCEs in fostering nurturing parenting behaviors among Asian American families. Although PCE measures may not fully capture culturally specific forms of positive experiences emphasized in Asian families [66,67], maternal PCE scores still emerged as an important correlate of favorable parenting behaviors in this study. This suggests that foundational positive relational experiences in childhood may exert a protective influence on parenting across cultural groups, even if their manifestations vary in form. In Asian American families, maternal PCEs may support nurturing parenting through culturally meaningful pathways, such as sustained parental involvement, commitment to children’s well-being, and relational responsibility, rather than solely through overt emotional expressiveness [66,79]. Thus, despite potential limitations in culturally specific measurement [79], our findings indicate that mothers’ positive early life experiences remain salient in shaping supportive parenting practices.

### 4.5. Child Physical Behaviors and Social-Emotional Development in Asian American Families

This pilot study found no significant differences in children’s MVPA across the three Asian ethnic groups. However, their average MVPA levels (69 min/day on average; 95% CI = 62, 76) were lower than the previously reported average of 79 min/day observed in a predominantly non-Hispanic White sample of 2-year-olds from the same geographic region [40]. Mean average acceleration metrics indicate lower PA volume among children of Indian Asian mothers than children of Filipino mothers (15.2 vs. 20.5 mg; effect size Cohen’s *d* = 0.73); however, the difference did not reach statistical significance, likely due to a small sample size.

Notable differences among the three groups emerged in screen time, sleep, and social-relationship development. Disaggregated data revealed that children of Filipino mothers exhibited greater challenges related to screen time and sleep. These differences may reflect variations in parental norms around technology use and daily routines, as well as broader sociocultural influences such as parental work schedules, household structure, and acculturation-related stress [80]. It is also possible that relatively higher ACEs and lower PCEs among Filipino mothers in this study may have contributed to greater perceived stress, which could influence parenting practices, including reliance on children’s screen media use as a coping strategy [81]. This, in turn, could affect sleep problems [82]. However, available data are extremely limited to support these potential mechanisms.

Across all three groups, average child–caregiver interaction T scores were above the reference norm. In particular, the children of Korean mothers demonstrated stronger child–caregiver interactions. While collectivistic values like interdependence and family cohesion are shared across Asian cultures, these stronger child–caregiver interactions in Korean families may stem from culturally rooted parenting practices that emphasize parental involvement and relational responsibility. For example, Korean mothers are reported to prioritize prosocial skills (e.g., sharing and helping), and often model these behaviors directly to their young children [83]. This proactive, example-driven approach may nurture strong relational bonds. These preliminary findings suggest the need for further explorations of protective relational factors that contribute to healthy socio-emotional development among Asian American children in future research. A full-scale study should replicate these findings and identify the underlying mechanisms.

### 4.6. Limitations and Future Directions

The use of a small, convenience-based sample and limited covariate adjustment warrant caution in interpreting the results. In particular, our findings are constrained by the inclusion of mothers with at least a decade of residence in the U.S., high education attainment, and living in high-resource neighborhoods, which may limit generalizability to more socioeconomically diverse or recently immigrated Asian American families. Nevertheless, these pilot data provide a critical foundation for generating hypotheses and refining the conceptual models that can be rigorously tested using robust causal inference methods in a future full-scale investigation. Importantly, the present study also offers preliminary theoretical and practical guidance for future large-scale epidemiologic surveys examining parenting and early life experiences among Asian American families. Future large-scale research should aim to include Asian American families from a broader range of immigration histories and socioeconomic backgrounds to enhance representativeness and capture heterogeneity.

The use of proxy-reported child development data could have introduced reporting bias. For example, mothers generally reported more favorable child outcomes than fathers, i.e., lower screen time, higher wellbeing, and more positive child–caregiver interactions. Moreover, many widely used developmental assessment tools have not been validated in Asian American populations. Future research should incorporate measurement validation to ensure the cultural relevance and accurate assessment of developmental outcomes. Lastly, as we observed challenges with wearable adherence, future research should integrate proactive engagement approaches and alternative wearable strategies to enhance compliance.

## 5. Conclusions

A remote study protocol was overall feasible; however, child compliance with hip accelerometer wear was suboptimal. Preliminary data revealed differences in child physical behaviors and social-emotional development across Asian ethnic groups. Future full-scale studies should enhance the engagement of socioeconomically diverse families and refine wearable data collection methods to improve data representativeness and completeness.

## Figures and Tables

**Table 1:** Participant characteristics.

	Asian Indian (n = 18)	Filipino (n = 12)	Korean (n = 18)
	Mean ± SD or n (%)	Mean ± SD or n (%)	Mean ± SD or n (%)
Children			
Age, months	2.1 ± 0.9	2.2 ± 1.2	2.0 ± 0.9
Sex: female	9 (50)	6 (50)	7 (39)
Speaking English only at home	7 (39)	10 (83)	8 (44)
COI: high or very high	15 (83)	9 (75)	15 (83)
Mothers			
Age, years	37 ± 3	35 ± 4	37 ± 4
Residential years in US, years	31 ± 10	31 ± 8	31 ± 8
Birth country: US	12 (67)	8 (67)	9 (50)
Education: ≥bachelor’s degree	17 (94)	12 (100)	18 (100)
Employed full-time	15 (83)	10 (83)	15 (83)
Fathers			
Age, years	38 ± 4	36 ± 4	37 ± 5
Race: Asian	10 (56)	0 (0)	11 (51)
Birth country: US	11 (61)	12 (100)	14 (78)
Education: ≥bachelor’s degree	18 (100)	10 (83)	15 (83)
Employed full-time	15 (83)	11 (92)	16 (89)

COI, Child Opportunity Index; SD, standard deviation.

**Table 2:** Descriptive statistics of child outcomes.

Variable	All (n = 48)	Asian Indian (n = 18)	Filipino (n = 12)	Korean (n = 18)	*p*-Value
	Mean ± SD	Mean ± SD	Mean ± SD	Mean ± SD	
Accelerometer wear, hours/day ^a^	13.7 ± 2.2	13.5 ± 1.9	14.7 ± 0.6	13.3 ± 2.8	0.32
Accelerometer-measured MVPA, minutes/day ^a^	69 ± 21	66 ± 24	76 ± 22	67 ± 19	0.56
Accelerometer-measured average acceleration, mg ^a^	17.6 ± 7.0	15.2 ± 5.9	20.5 ± 8.4	17.6 ± 6.6	0.27
Screen time	36 ± 46	30 ± 29	53 ± 71	30 ± 37	0.34
PROMIS EC Sleep Problem T-score	50 ± 9	48 ± 10 ^b^	55 ± 5 ^b^	49 ± 8	0.10
PROMIS EC Positive Affection T-score	50 ± 9	50 ± 10	50 ± 8	51 ± 9	0.85
PROMIS EC Curiosity T-score	50 ± 7	52 ± 7	47 ± 9	50 ± 6	0.15
PROMIS EC Persistence T-score	47 ± 7	47 ± 6	46 ± 8	49 ± 7	0.46
PROMIS EC Flexibility T-score	51 ± 8	51 ± 9	52 ± 9	50 ± 8	0.87
PROMIS EC Frustration Tolerance T-score	48 ± 8	48 ± 11	48 ± 6	48 ± 6	0.99
PROMIS EC overall well-being T-score ^c^	49 ± 5	49 ± 6	48 ± 5	50 ± 4	0.78
PROMIS EC Parent–Caregiver Interaction T-score	54 ± 9	52 ± 9 ^d^	52 ± 10 ^d^	58 ± 6 ^d^	0.05

MVPA, moderate- and vigorous-intensity physical activity; PROMIS EC, Patient-Reported Outcome Measurement Information System Early Childhood; SD, standard deviation. ^a^ Accelerometer data sample size n = 10 for the Asian Indian group, n = 9 for the Filipino group, and n = 15 for the Korean group. ^b^ ANOVA ad hoc Tukey test *p*-value < 0.10 between Asian Indian and Filipino groups. ^c^ PROMIS EC overall well-being score was calculated by averaging PROMIS EC Positive Affection, Curiosity, Persistence, Flexibility, Frustration, and Tolerance scores. ^d^ ANOVA ad hoc Tukey test *p*-value < 0.05 between Asian Indian and Korean groups and between Filipino and Korean groups.

**Table 3:** Descriptive statistics of mothers’ experiences and parenting.

Variable	All (n = 48)	Asian Indian (n = 18)	Filipino (n = 12)	Korean (n = 18)	*p*-Value
	Mean ± SD	Mean ± SD	Mean ± SD	Mean ± SD	
Experiences					
Adverse childhood experience (0–10), n	1.5 ± 1.7	1.4 ± 1.7	2.4 ± 1.6 ^a^	1.0 ± 1.6 ^a^	0.08
Positive childhood experience (0–7), n	5.6 ± 1.6	6.1 ± 0.9 ^b^	4.4 ± 2.1 ^b^	6.0 ± 1.3 ^b^	<0.01
Acculturation score	3.2 ± 0.7	3.1 ± 0.6	3.5 ± 0.6	3.1 ± 0.7	0.20
Everyday discrimination score	1.9 ± 0.6	1.9 ± 0.6	2.0 ± 0.6	2.0 ± 0.6	0.80
PARYC Parenting Practice score					
PARYC-supporting positive behavior	5.7 ± 0.6	5.9 ± 0.5	5.5 ± 0.5	5.7 ± 0.8	0.20
PARYC—setting limits	5.1 ± 1.0	5.2 ± 1.1	4.8 ± 0.9	5.2 ± 1.1	0.47
PARYC—proactive parenting	4.8 ± 1.4	4.8 ± 1.4	4.4 ± 1.4	4.9 ± 0.9	0.66
PARYC—overall parenting score ^c^	5.2 ± 0.8	5.3 ± 0.9	4.9 ± 0.9	5.3 ± 0.8	0.43
		n (%)	n (%)	n (%)	
Frequency of Positive Parenting Practices					
Read to child ≥ 4 days/week	42 (88)	14 (78)	12 (100)	16 (89)	0.19
Tell stories or sing songs to a child ≥ 4 days/week	38 (79)	14 (78)	10 (83)	14 (78)	0.92
Family meal ≥ 4 days/week	39 (81)	12 (67)	11 (92)	16 (89)	0.13

PARYC, Parenting Young Children scale; SD, standard deviation. Acculturation score 1 = low acculturation (high Asian identification) to 5 = high acculturation (high western identification); everyday discrimination score 1 = never to 6 = almost everyday. ^a^ ANOVA ad hoc Tukey test *p*-value < 0.10 between Filipino and Korean groups. ^b^ ANOVA ad hoc Tukey test *p*-value < 0.05 between Asian Indian and Filipino groups and between Korean and Filipino groups. ^c^ PARYC parenting practice score was calculated by averaging PARYC-supporting positive behavior, proactive parenting, and setting limits scores.

**Table 4:** Multivariable mixed-effects models for child development outcomes.

Predictor	Screen Time (Minutes/Day)	PROMIS EC Sleep Problem T-Score	PROMIS EC Wellbeing T-Score	PROMIS EC Child–Caregiver Interaction T-Score
	β ± SE	β ± SE	β ± SE	β ± SE
Mother vs. Father respondent	−8.9 ± 5.4	−0.8 ± 1.0	1.4 ± 0.8 *	3.1 ± 1.2 **
Filipino vs. Asian Indian	26.1 ± 14.7 *	6.3 ± 2.6 **	−0.7 ± 1.3	1.3 ± 2.4
Korean vs. Asian Indian	11.0 ± 13.3	3.0 ± 2.4	0.3 ± 1.2	7.2 ± 2.1 ***
Male sex	1.0 ± 12.0	−1.9 ± 2.2	−0.3 ± 1.1	−0.3 ± 2
Child age in years	12.4 ± 6.3 *	2.1 ± 1.1 *	0.04 ± 0.6	0.5 ± 1.0
PARYC—overall parenting practice scores	4.5 ± 4.8	−3.0 ± 0.9 ***	2.4 ± 0.6 ***	3.4 ± 0.9 ***

* *p* < 0.10; ** *p* < 0.05; *** *p* < 0.01; β, coefficient; PROMIS EC, Patient-Reported Outcome Measurement Information System Early Childhood; SE, standard error. Multivariable mixed-effect models used both mother- and father-reported data (n = 96); PARYC, Parenting for Young Children scale.

## Data Availability

The datasets used during the current study are available from the corresponding author on reasonable request.

## Supplementary Materials

The following supporting information can be downloaded at https://www.mdpi.com/article/10.3390/children13020256/s1. Figure S1. (a). Maternal adverse childhood experiences (ACEs) and (b). Maternal positive childhood experiences (PCEs). Table S1. Child age-adjusted association between adverse childhood experiences (ACEs) and positive childhood experiences (PCEs) and parenting practices among Asian mothers.

## Abbreviations

The following abbreviations are used in this manuscript:

ACE	adverse childhood experience
COI	Child Opportunity Index
MVPA	moderate- and vigorous-intensity physical activity
PA	physical activity
PARYC	parenting young children scale
PCE	positive childhood experience
PROMIS EC	Patient-Reported Outcome Measurement Information System Early Childhood
