# Supplementary material for: Early Childhood Behavioral and Social-Emotional Development Among Asian Indian, Filipino, and Korean Families in the United States: A Pilot Study
Source: Children (Basel). 2026 Feb 12;13(2):256. doi: 10.3390/children13020256 (PMC12939503; doi:10.3390/children13020256)
Supplement: Supplementary file 1 [file children-13-00256-s001.zip › children-4136234-supplementary.pdf]

Supplementary Data

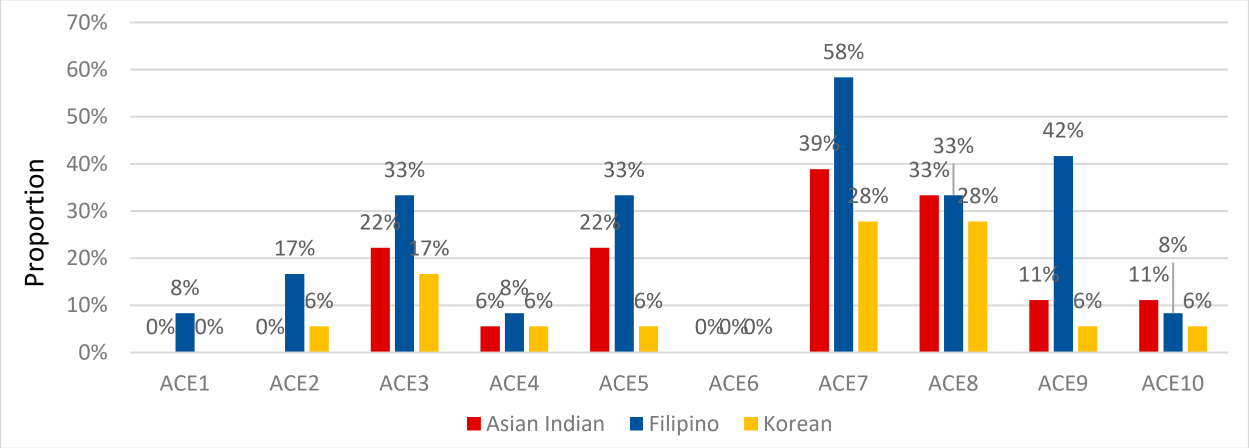

a. Maternal adverse childhood experiences (ACEs).

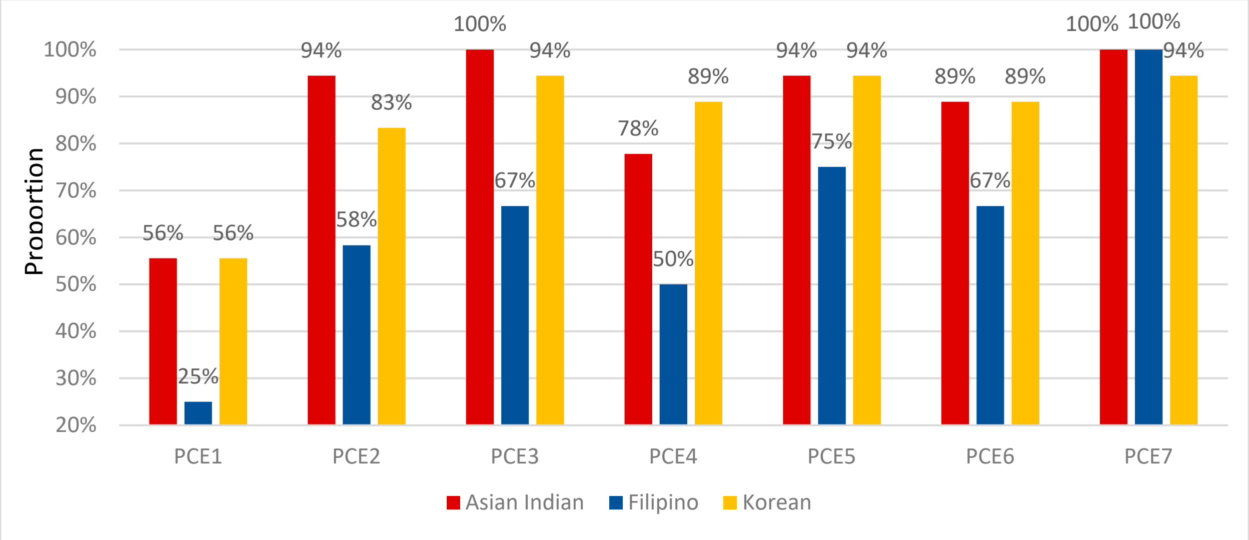

b. Maternal positive childhood experiences (PCEs).

Supplementary Figure S1. a. Maternal adverse childhood experiences (ACEs) and b. Maternal positive childhood experiences (PCEs).

ACE1, did not have enough to eat; **ACE2**, lost a parent through divorce, abandonment, death, or other reason; **ACE3,** lived with one depressed, mentally ill, or attempted suicide; **ACE4**, lived with one with a drinking or drug problem; **ACE5,** had an adult at home who hit, punched, beat, or threatened to harm each other; **ACE6**, lived with one who went to jail; **ACE7**, had an adult in your home who swore at you, insult you, or put you down; **ACE8**, had an adult at home who physically hurt you in any way; **ACE9**, felt that no one in your family loved you or thought you were special; **ACE10**, experienced unwanted sexual contact; **PCE1**, felt comfortable talking to your family about feelings; **PCE2**, sensed that your family was supportive; **PCE3,** enjoyed participating in community traditions; **PCE4**, felt sense of belonging in high school; **PCE5,** felt supported by friends; **PCE6**, had at least two non-parent adults who cared for me; **PCE7,** felt safe and protected by an adult in your home.

Supplementary Table S1. Child age-adjusted association between adverse childhood experiences (ACEs) and positive childhood experiences (PCEs) and parenting practices among Asian mothers.

	PARYC-supporting positive behavior	PARYC-proactive parenting	PARYC-Setting limits	PARYC-overall parenting score
	β±SE	β±SE	β±SE	β±SE
Child age	0.36±0.13***	0.24±0.10**	0.46±0.20**	0.40±0.15***
ACE	-0.003±0.1	0.06±0.05	-0.06±0.10	-0.01±0.08
PCE	0.14±0.09	0.11±0.06*	0.06±0.13	0.23±0.10**

*p<0.10; **p<0.05; ***p<0.01; β, coefficient; SE, standard error. PARYC, Parenting Young Children scale.
